# Liver damage in the context of SARS-CoV-2. Covid-19 treatment and its effects on the liver

**DOI:** 10.25122/jml-2022-0177

**Published:** 2022-06

**Authors:** Denisa Marilena Săbiescu, Adina Maria Kamal, Constantin Kamal Kamal, Dragos Ovidiu Alexandru, Paul Mitruț

**Affiliations:** 1 Department of Internal Medicine, University of Medicine and Pharmacy of Craiova, Craiova, Romania; 2 Department of Family Medicine, University of Medicine and Pharmacy of Craiova, Craiova, Romania; 3 Department of Informatics and Biostatistics, University of Medicine and Pharmacy of Craiova, Craiova, Romania

**Keywords:** COVID-19, hepatotropism, liver biochemistry, liver disease, SARS-COV2

## Abstract

Since COVID-19 was declared a pandemic by the World Health Organization, the scientific community has tried to protect the population from the infection and its effects through multiple lines of evidence. Patients at high risk of developing severe disease were advised to protect themselves and practice effective physical distancing. Phenotypes specific to this infection need to be reviewed to understand COVID-19 and its clinical manifestations. When the pandemic began, the scientific community was concerned with the unfavorable outcome of cases with pre-existing liver disease. There have been speculations about risk factors for severe diseases such as liver disease, age, gender, and association with obesity or diabetes.

## INTRODUCTION

In December 2019, Wuhan, China, reported the first case of the RNA virus known as severe acute respiratory syndrome coronavirus 2 (SARS-CoV-2). Since then, the virus quickly spread worldwide, with devastating effects on health [1]. Most people with SARS-CoV-2 infection exhibit mild symptoms, such as fever, coughing, anosmia, and headache. Within 10 days, about 15% of those who encounter any of these may undergo severe protracted involution, which can cause coagulopathy, late respiratory compromises, multi-organ failure, and death [2, 3]. Critically ill patients continue to have higher fatality rates despite standard care practices, including oxygen supplementation, involuntary ventilation, and many supportive interventions. Risk factors linked with critical COVID-19 include age, male sex, and comorbidities, such as diabetes, heart disease, arterial hypertension, and malignancies [4, 5]. Immunomodifying and direct antiviral agents, as well as targeted therapeutic approaches against infection, are still being evaluated in clinical trials. Understanding patient cohorts that require quick therapeutic interventions is an important clinical goal [6, 7]. Additionally, the development of the SARS-CoV-2 vaccine advanced remarkably, with the top candidates presenting extremely encouraging data on its safety and efficacy from phase III tests. There is currently unprecedented demand for vaccine deployment worldwide; thus, it is necessary to identify which patients are more susceptible to the negative effects of COVID-19 to determine priority in immunization programs.

Pre-existing chronic liver disease (PELD) is a critical condition that increases the likelihood of poor outcomes after contracting SARS-CoV-2 [8]. This is especially true given the possibility that COVID-19 and severe chronic liver disease (CLD) are risk factors, as well as age, obesity, and diabetes. A substantial advancement in COVID-19 may also be attributed to the association of advanced liver disease with compromised immunity and coagulopathy [9, 10]. With approximately 122 million people worldwide suffering from liver cirrhosis, of which 10 million have decompensation disease, CLD has a significant global impact [11]. It is crucial to comprehend the natural evolution of COVID-19 in patients with CLD, spanning various etiologies and the specter of severity of the liver injury.

This paper is based on reviewing the pathophysiology and impact of SARS-CoV-2 infection in patients with pre-existing liver disease (PELD) and on rapidly collecting data from worldwide cohorts throughout 2020. In addition, we examined the data supporting direct infection of SARS-CoV-2 in the liver cells and looked into putative mechanisms causing SARS-CoV-2-related liver damage. As a predictor during COVID-19, we also looked at liver biochemistry. Finally, we draw attention to the important effects of pandemics on future patient behavior and hepatology services, potentially increasing the frequency and severity of liver disease.

## SARS-COV-2 HEPATOTROPISM

According to an analysis of RNA sequencing in healthy livers, cholangiocytes (similar to type 2 alveolar cells) express ACE2 at the highest levels, followed by sinusoidal endothelial cells and hepatocytes [10, 11]. In several kinds of liver cells, TMPRSS2 and FURIN displayed a diverse pattern of gene expression [10]. Despite this, only a small number of hepatocytes co-expressed TMPRSS2 and ACE2 in a combined analysis of three sets of data sequencing an RNA unicellular liver tissue from healthy persons [12]. Therefore, it is crucial to know how different liver cells respond to SARS-CoV-2 infection using cellular and organoid experimental models. Huh-7 and HepG2 cell lines generated from hepatocellular carcinoma are capable of supporting the whole viral life cycle [13]. Despite all of these, replication has not yet been established in primary hepatocytes. The contrast between the cellular models may be explained by mutations related to cancer in hepatic cells, such as mutations in the tumor suppressor p53, which works to inhibit replication under normal circumstances [14].

Bile duct epithelium may enable the development of pseudo-particles, according to Zhao and colleagues, who created human hepatic ductal organoids that express ACE2 and TMPRSS2 [15]. SARS-CoV-2 could engage in the replication of cholangiocytes to a minimum, in vivo experiments, without leading to cellular demise [16]. This approach is congruent with other long-term replication reservoirs of the virus, such as those found in the small bowel, which could benefit cell responses to the virus [17]. Additionally, it was revealed that hematopoietic organoids made from pluripotent human stem cells, principally hepatocytes expressing albumin and ACE2, can allow the entry of SARS-CoV-2 pseudo particles [15].

The real impact of liver injury and liver disease due to SARS-CoV-2 hepatotropism is unclear, and no study has yet specifically explored the histological changes found at pacients with pre-existing COVID-19 and CLD. From the beginning of COVID19, some studies revealed more than 30-times enhanced ACE2 expression in the liver of pacients with hepatitis C virus-linked cirrhosis compared to healthy persons [18]. Additionally, not only patients with steatosis but also those with obesity and non-alcoholic steatohepatitis had their TMPRSS2 and RNm hepatic ACE2 expression altered [19]. In line with hypoxic markers, patterns of hepatic infection in rodents linked to biliary route ligation were linked to increased hepatic ACE2 expression and activity [18, 20]. Since ACE2 was discovered as a gene capable of inducing interferon in respiratory epithelial cells, liver lesions and infections may increase SARS-CoV-2 hepatotropism by adjusting viral receptor expression [21, 22]. Due to the possibility that the truncated variant of ACE2, known as delta ACE2, rather than the viral receptor molecule itself, is up-regulated, this statement should be read cautiously [23]. Although factors with tissue-specific regulating infection with SARS CoV2 were not fully apprehended, the importance of additional accommodative receptors in the viral entry is becoming better acknowledged. Evidence suggests that the ACE2-dependent coronavirus activation *in vitro* and infection with hepatitis C are made possible via the high-density lipoprotein scavenger receptor B type 1 (SR-B1). Additionally, SR-B1-targeting treatments decreased the mediated lipoprotein increase of SARS-CoV-2 infection [24, 25]. In addition, the histological evaluation of the study's liver tissue revealed that ACE2 was only sporadically expressed in the liver.

It is technically and clinically difficult to get a liver biopsy and identify infection with the virus during the brief period of acute respiratory sickness. Extrapulmonary infection remains discernible, especially in the gastrointestinal system, with SARS-CoV-2 PCR in feces lingering positive for up to 7 days after lung clearance [26]. General enterocyte infection has also been extensively studied [27, 28], and even after treating a clinical infection, viral proteins and viral RNA can still be found in intestinal biopsies for several months [16]. In situ hybridization of SARS-CoV-2 was found in 68% of biopsy samples from 48 patients who did not survive severe COVID-19 [29]. The histological evaluation also revealed vascular abnormalities, including moderate portal-cava inflammation (66%) and portal-cava fibrosis (100%) as well as portal-cava venous and sinusoidal micro thrombosis (50%) and micro-and macrovesicular steatosis (50%) (60%) [30]. The most recent discovery may point to some fundamental underlying liver illness and is most likely related to non-alcoholic fatty liver disease (NAFLD) since some risk factors, such as hypertension and cardiovascular conditions, were also present in this sample. Again, given the level of expression of the viral entry receptor of cholangiocytes, the absence of histological evidence of biliary injury is clear. Large maps of protein interaction were found to connect the main protein of SARS-CoV-2, NSP5, and mitochondrial components, which is compatible with this finding. On the other hand, there was no evidence of active viral replication in the liver according to a deep proteomic study of postmortem tissue from 19 COVID-19 patients [31, 32]. However, the signatures of liver proteins indicated upregulated profibrotic pathways, deregulated oxidation of fatty acids and oxidative phosphorylation, and markers of immune activation. This changing proteomic landscape was associated with multi-organ injuries, steatosis, and necrosis of clotted hepatocytes [32].

## COVID-19 AND LIVER CHEMISTRY

### Pattern and prevalence of liver function test abnormalities in COVID-19

Even though the exact impact of COVID-19 on the liver is unknown, biochemistry abnormalities are widespread in COVID-19 patients, who make up around 15–65% of those with SARS-CoV-2 infection [33–41]. Abnormalities of liver biochemistry in COVID-19 are generally characterized by slight (1-2 times the superior limit of normal) increases in alanin-aminotransferaze (ALT) aspartat-aminotransferaze (AST) levels, reported in approximately 40–60% of pacients. Furthermore, hypoalbuminemia, a nonspecific marker of disease severity, was reported in 36–38% of cases [42] associated with severe outcomes of COVID-19 [43]. However, severe liver lesions, increased serum bilirubin levels, and synthetic liver dysfunctions are uncommon in pacients with COVD-19 [39, 40, 42]. Regardless of pre-existing liver illness status, liver biochemistry abnormalities are detected at equal rates [44–46].

### Causes of increase in liver enzymes in COVID-19

The elevated levels of liver enzymes in COVID-19 may be caused by a variety of factors. Patients with SARS-CoV-2 have shown nonspecific liver biopsy findings, such as steatosis, moderate lobular and/or inflammation of the portal vein, and pathology of the vascular system [29, 45, 46]. The cause of liver function test abnormalities is probably multifactorial, with possible involvement from inflammatory response, extrahepatic cause of transaminases increase, drug-induced liver disease, and direct virological effect on liver cells.

Patients hospitalized with COVID-19 experience an increase in blood aminotransferase (AST) levels that is favorably correlated with alanine aminotransferase (ALT) levels but not with markers of muscle breakdown such as creatine kinase (CK) or systemic inflammation (C reactive protein and ferritin) [47, 48]. These suggest that high liver enzymes in COVID-19 result from direct liver damage, although rhabdomyolysis associated with COVID-19 is not frequently discovered [49–51]. In addition to some circumstances, such as alcohol-associated liver disorders, any drug-induced liver harm (such as lamotrigine), ischemic hepatitis, and cirrhosis, it is frequently observed that AST surpasses ALT during COVID-19 [48]. Although the causes of the increase in aminotransferases predominance AST are not yet fully understood, they could be mitochondrial dysfunction associated with COVID-19 [31], hepatic steatosis induced by SARS-CoV-2 and impaired liver perfusion due to microthrombotic disease [29, 50]. The prevalence of liver vein thrombosis in COVID-19 was reported at 29% in a systematic analysis [51]. Intriguingly, a rise in AST with other viral pneumonia, such as the H1N1 influenza infection, where levels are elevated simultaneous with decreasing peripheral oxygen saturation, suggests that systemic hypoxia may play a contributing role in COVID-19 [52, 53]. SARS-CoV-2 is associated with a systemic infection that, like many other infections, is expected to enhance liver biochemistry by producing cytokine [54, 55]. Patients with significantly increased serum LT levels frequently have high liver-produced CRP, D-dimers, ferritin, and IL-6 levels [42, 43, 54–56].

The main factor causing CRP generation and high levels of IL-6, which are connected to liver damage in COVID-19, is IL-6 generated by T cells, macrophages, and monocytes in response to the activation of the inborn and adaptive immune systems [42, 54]. In particular, the level of IL-6 rises during COVID-19 illness, falls as patients recover, and is correlated with the severity of the disease.

There are few possible factors contributing to the modified biochemistry of the liver in COVID-19, including ischemic hepatitis, liver congestion related to cardiovascular damage, and the increase of transaminases from causing the breakdown of skeletal and cardiac muscle [49]. Venous thromboembolism and arterial thrombosis are known characteristics of COVID-19 [57–60], including the liver [29–46], that may contribute to an increase in liver biochemistry. Finally, a drug-induced liver injury could contribute to an increase in liver enzymes and could have been more usual at the beginning of the pandemic because of the use of experimental therapies [61, 62]. However, the pattern of liver function tests observed in research during the pandemic has not been extensively mapped yet. Lopinavir, Ritonavir, Tocilizumab, and Remdesivir are specific COVID-19 treatments that have been linked to cases of drug-induced liver damage. Remdesivir's hepatotoxicity has been disputed [63–65]. WHO safety reports still reveal a significant statistical risk for hepatic harm with Remdesivir, despite randomized studies in COVID-19 demonstrating similar elevations in liver enzyme levels between treatment and control groups [66, 67]. After carefully reviewing the WHO safety data, we uncovered an increased risk of developing liver damage associated with Remdesivir [67]. However, these findings may not have clinical relevance since the SOLIDARITY study did not show any utility of Remdesivir in patients hospitalized with COVID-19 [68].

### Elevated liver enzymes and prognostic value

There is an ongoing disagreement over the predictive value of increased liver enzymes in individuals infected with SARS-CoV-2. According to certain studies, elevated liver enzyme levels are linked to adverse manifestations such as shock, admission to an intensive care unit (ICU), and mechanical ventilation [41, 63, 69–72].

There have been contradictory opinions about liver enzymes involvement in mortality and morbidity prediction, as some have discarded the hypothesis that elevation in liver function tests may have any predictive value [69, 73], and others proved that elevation over 5 times the upper limit is strongly correlated with high mortality risk [39, 42, 74, 75]. It was suggested that patients with severe disease might respond more robustly to intensive treatments and prognostic indicators of higher liver enzyme levels [76].

## SARS-COV-2 INFECTION AND LIVER DISEASE

Patients with varying degrees of liver disease, especially those with liver cirrhosis, have increased risk of infections due to an abnormal inflammatory response. This dysfunction is known as cirrhosis-associated immune dysfunction (CAID). Reduced complement system components, the activation of macrophages, impaired neutrophil and lymphocyte function, positive regulation of toll-like receptors, and gut dysbiosis are all examples of immunological dysfunction [7, 77]. It was established that CLD predisposes to various viral or fungal diseases [78]. although attention was mainly focused on the mechanisms causing severe bacterial infections [79]. Studies from large cohorts of patients with COVID-19 and population observations using health records do not indicate that patients with chronic liver disease are overrepresented [4, 35]. In fact, data from the US medical records show that patients with cirrhosis have a smaller risk of being tested positive for SARS-CoV-2 infection [80, 81]. Cirrhosis does not appear to offer protection against SARS-CoV-2 infection; therefore, the reduced percentage of positive tests is presumably the result of better adherence and repeated testing.

The etiology of liver disease may influence the clinical result of COVID-19. However, in these studies, patients were not diagnosed with nonalcoholic fatty disease (NAFLD), partly because liver steatosis was not documented or alcohol intake was not evaluated. Aging, high body mass index, and a history of diabetes are important in influencing the morbidity and mortality of the general population [6]. There are significant discrepancies within the body of data on the impact of NAFLD due to the COVID-19 course. These discrepancies might be attributed to challenges in distinguishing the impact of nonalcoholic fatty liver disease from other metabolic comorbidities, from the origin of virus-induced steatosis or various diagnostic criteria. Today, the world hepatology associations are faced with reclassifying NAFLD as liver disease linked to metabolic dysfunction, making the last of these issues of special importance [82–84]. A retrospective study of 202 patients with SARS-CoV-2 found that NAFLD was a risk factor for developing COVID-19, having increased liver enzyme levels, and taking much more time to remove the virus [85]. Another study of 327 patients identified a link between NAFLD and the incidence of critical COVID-19 among patients under 60 [86, 87]. In a previous study, steatosis was not associated with death [88], later confirmed in a large international study covering 29 countries [89].

Similarly, 287 patients positive for SARS-CoV-2 who underwent MRI evaluation showed that those with obesity and liver fat percentage of >10 had twice the risk of having symptomatic COVID-19 [89]. Steatosis was seen in 43% of 155 consecutive COVID-19 hospitalized patients, although it was not independently correlated with death [89]. The death rate for individuals with NAFLD was 1.01 in a large worldwide cohort of 745 patients with pre-existing liver disease (PELD) from 29 countries monitored using the SECURE – Cirrhosis and COVID-19 records (95% CI 0.57–1.79) [89]. The only cause of liver disease in the same research with a meaningful odds ratio for mortality was alcohol-related liver disease 1.79 (95% CI 1.03–3.13) [89]. Although immunosuppression was used in 86% of the cases, registry data for 70 patients with autoimmune hepatitis associated with positive infection of SARS-CoV-2 revealed similar outcomes to those of patients with high-grade aetiological CLD and propensity score controls [90]. In several series, COVID-19-induced lung illness was an important cause of death in CLD patients, followed by liver death [89, 91].

**Figure 1:**
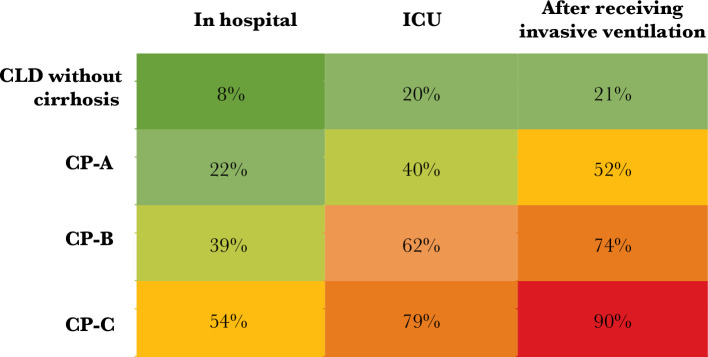
The initial stage of liver illness and amount of medical assistance as a predictor of mortality after SARS-CoV-2 infection. Rate fatality in patients with severe acute respiratory distress syndrome (SARDS) after hospitalization, at intensive care units (ICUs), and with invasive ventilation, separated by the stage of liver affection. Child-Pugh, or CP. derived from [89], CC BY 4.0 (https://creativecommons.org/licenses/by/4.0/).

Following infection with SARS-CoV-2, morbidity and mortality in patients with pre-existing liver disease increase with the degree of cirrhosis, according to the Child-Pugh classification (CP). Even though the percentage of patients hospitalized in the SECURE – Cirrhosis and COVID-Hep records made no difference between patients with CLD and CP class B and C, a high incidence of ICU admissions and renal transplant was observed, artificial ventilation, and even death. Additionally, there was a higher death rate for all patients as a result of their need for more intense medical care, with those classified as CP-C having a minimal survival rate (10%) following mechanical ventilation (Figure 1). After initial characteristics were taken into account, the degree of pre-existing liver cirrhosis was strongly associated with COVID-19-linked mortality, and the risk of death increased as the cirrhosis progressed [89]. Although a significant initial mortality rate is linked with COVID-19 in patients with cirrhosis, death and rehospitalization rates at 3 months are comparable to those in patients with liver cirrhosis only [92]. Therefore, SARS-CoV-2 infection does not appear to accelerate the progression of liver disease over the natural course of cirrhosis once the acute infectious phase has passed. Acute-on-chronic liver failure (ACLF) can be brought on by COVID-19. Viral illnesses can also cause CLF, although more frequently linked to bacterial infections [83, 93], characterized by an increase in the severity and frequency of extrahepatic organ failure and liver-specific decompensation.

The severe pulmonary illness COVID-19 and the CLF are likely to interact at the base of chronic liver disease. The immune system's response to infection is amplified by cirrhosis due to an increase in early endotoxemia and cytokine production. This situation can be specifically severe in pacients with alcohol-induced hepatic disease [94–96], emphasizing the potentially high mortality in this cohort [89].

## MORTALITY

The gut microbiota's composition influences the form of COVID-19, which could influence the gut's immunological responses [97]. Given that intestinal permeability and the composition and function of the bowel microbiota are both affected by cirrhosis of the liver [98, 99], changes in the axial intestinal may contribute to a severe evolution of COVID-19 observed late in this group of pacients. However, additional research is needed about the mechanisms that stand at the base of COVID-19.

Finally, the COVID-19 pandemic exposed the long-established associations between race, socioeconomic status, and negative health outcomes. This problem affects patients with CLD with an increased risk of SARS-CoV-2 infection due to socioeconomic disadvantage in their communities [100, 101]. The use of telemedicine, particularly video technology, is further hampered by racial and socioeconomic gaps in internet access, particularly among patients with pre-existing liver illness [100].

### Managing patients with COVID-19 and concomitant hepatic illness in detail

The ideal treatment for patients with subacute liver injury infected with SARS-CoV-2 is still under development. However, treatment strategies for this patient population were improved by examining COVID-19 progression through multicenter and world cohorts, as described in multiple consensus guidelines [101–103].

First, it is critical to understand that cirrhotic individuals are especially susceptible to severe consequences due to COVID-19. In patients with cirrhosis, hepatic decompensation is likely the initial and sole sign of SARS-CoV-2 infection, with just 24% exhibiting concurrent pulmonary symptoms. Importantly, patients with autoimmunity hepatitis have mortality rates linked to COVID-19 that are similar to those of the general matched population [90, 104, 105]. Additionally, using immunosuppression does not increase one's chance of dying. These results should comfort medical professionals and offer a convincing justification for delaying normal immunosuppression for patients during the COVID-19 treatment. The initial prognosis for patients with COVID-19 and advanced cirrhosis is bleak, with a death rate of up to 80% and a requirement for extensive therapy support [89].

Respiratory failure is the primary cause of mortality in individuals with cirrhosis and COVID-19; however, the processes underpinning this result are yet unknown. Regular thromboprophylaxis is always advised for hospitalized patients with COVID-19; cirrhosis and coagulopathy may increase the risk of thrombotic complications [101].

Studies on the COVID-19 pandemic indicated a benefit of anticoagulation in lowering port pressure, while it is still uncertain if enhanced venous thromboembolic prophylaxis will be helpful for this group of patients [106, 107] with minimal danger of severe hemorrhage [108]. Additionally, a multicenter trial in northern Italy found that using thromboprophylaxis to treat 40 patients with cirrhosis diagnosed with COVID-19 did not result in any significant hemorrhagic complications. Unfortunately, patients with severe cirrhosis are not included in many studies investigating optimal thromboprophylaxis after hospitalization with SARS-CoV-2 infection [109, 110].

The rush to create targeted treatments for COVID-19 is still going strong. Patients with cirrhosis are much less likely than those without to receive specific antiviral therapy, according to a global registry research from 29 countries and 130 different institutions (33% *vs*. 52%; P=0.001) [89]. This may be due to worries about the hepatotoxicity of the medication.

## COVID-19 IN LIVER CARE

**Figure 2:**
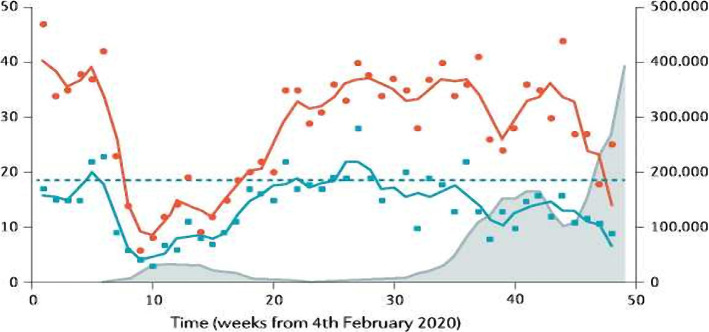
Activity of liver transplant in the UK before and during the COVID-19 pandemic. Data from the Blood and Transplant Service at the UK National Health Service.

From the beginning of the COVID-19 pandemic, SARS-CoV-2-infected patients were the focus of prevention, control, and care; as a result, it was reasonable to cut back on and improve services for non-emergent medical conditions. Unfortunately, such regulations invariably adversely impact patients, especially those with CLD. Morbidity and mortality will rise over time due to delayed diagnosis and treatment of numerous liver disorders [111] (Figure 2).

### Cirrhosis

The fundamental liver disease, hepatocellular carcinoma (HCC), varicose veins, and the early detection and tracking of cirrhosis complications are all important in individuals with cirrhosis [104, 112]. During a pandemic, all of these strategies may be impacted. A decompensation episode can result from, for instance, delaying the start of antiviral therapy in patients with chronic viral hepatitis and relapsing alcoholism [113].

### Hepatocellular carcinoma

Currently, the European Association for the Study of the Liver (ESL) and About SLD support continuing hepatocellular carcinoma (HCC) treatment in patients at increased risk (such as those with severe cirrhosis or chronic hepatitis with B virus) during COVID-19.

### Eliminating viral hepatitis

The WHO established a target to eradicate hepatitis with virus B or C as a significant danger to world health by 2030 in its first Global Report on Hepatitis published in 2017 [114]. Since then, medical service providers, contributors, and patients have teamed up to increase viral hepatitis screening, diagnosis, evaluation, and treatment. Although different countries adopted measures during the COVID-19 pandemic, such as telemedicine and the auto administration of medicines, to assure a continuous antiviral therapy, there has been a serious impact on identifying new cases and initiating tratament. According to modeling research conducted for 110 countries, a delay of one year in eradicating hepatitis will result in an increase of 44,800 and 72,300 deaths from HCC and hepatitis, respectively. Additionally, strategies for creating testing, tracking contacts, and administering vaccinations are becoming much more commonplace in the governmental and healthcare systems. There may be a chance to improve this substructure to fight chronic viral hepatitis at the population level [115].

Major research failures occurred at institutions worldwide, including the closure of libraries, the redirection of funds to COVID-19 investigations, and the loss of cell cultures during nationwide lockdowns [116].

## CONCLUSION

In the context of primary assistance medical care, liver injury offers advance warning about imminent critical disease. Early detection and prompt referral can play a crosscutting role in managing morbidity and rates of death in COVID-19.

Abdominal ultrasound is also essential for all patients who test positive for SARS-CoV-2, in conjunction with blood investigations.

Due to the unknowns surrounding infection control and the challenges of adhering to study guidelines, COVID-19 significantly impacts liver research, particularly clinical trials. In addition, circumstantial restrictions and ill patients can obstruct the tracking, evaluation, and dissemination of research. Due to these factors, several sponsors have ceased enrolling new patients in clinical trials, which has the potential to stall the crucial development of novel medications.

The consequences of SARS-CoV2 infection on liver function became important in COVID-19, especially in patients with pre-existing cirrhosis, who are at increased risk of disease progression or increased mortality. While additional studies are needed to understand the pathogenic mechanisms that lead to this clinical deterioration, there may be contributions from systemic inflammatory response, coagulability disorders, and immune dysfunction.

Several studies tried to determine the particular hepatotropism of SARS-CoV-2. However, the clinical results of direct virological infection of hepatic cells are still not determined.

Immune dysfunction pairing with liver cirrhosis has a much more critical effect on the development of COVID-19 than pharmacological immunosuppression. With the effective vaccines currently available for SARS-CoV-2 infection, patients with cirrhosis should be regarded as the first ones for immunization, and the medical staff should be prepared to take notice of the immune results in this subpopulation.

Finally, we must all be concerned about the many negative effects that the pandemic had and will have on medical services and the unhealthy behaviors of patients, which could culminate in a high amount of the global burden of liver disease in the coming months and years. Furthermore, no one should undervalue the negative impact of the pandemic on current fundamental non-COVID-19 and translational research.
